# OMRT-15. Multidisciplinary management of non-small cell lung cancer patients with leptomeningeal metastasis in the TKI era

**DOI:** 10.1093/noajnl/vdab070.039

**Published:** 2021-07-05

**Authors:** Kuan-Yu Chen, Abel Po-Hao Huang

**Affiliations:** 1 National Taiwan University, Department of Medicine, Taipei, Taiwan; 2 National Taiwan University Hospital, Department of Surgery, Neurosurgery, Taipei, Taiwan

## Abstract

**Background:**

leptomeningeal metastasis (LM) is a devastating scenario in patients with non-small cell lung cancer (NSCLC), with an estimated median overall survival (OS) of 4–6 months from diagnosis. Several studies have clarified the prognosis of treatment modalities after LM. However, just a few studies have clarified the prognosis of LM patterns. We evaluate the prognosis based on various patterns of LM under multidisciplinary treatment (MDT).

**Method:**

This retrospective study evaluated NSCLC patients treated at National Taiwan University Hospital between 2007–2019 with brain metastases (BM) and LM. LM was classified into LM only, LM concurrent with BM, and LM after BM. Treatments including systemic therapy, whole-brain radiotherapy (WBRT), stereotactic radiosurgery (SRS), and intrathecal chemotherapy with Methotrexate (IT MTX) were recorded. BM excision was done by a neurosurgeon using minimally invasive neurosurgery. The MDT was done according to patients’ clinical situations. Kaplan-Meier methodology was used to describe overall survival OS. Multivariate Cox regression model was used to access prognostic factors.

**Result:**

One hunderd patients with NSCLC CNS metastasis was included in this study. Median OS in patients with single, oligo and multiple BM was 42.0 months (95% CI= 0.12–83.89), 58.1 months (95% CI= 13.00–103.26), and 21.3 months (95% CI= 16.93–25.73), respectively. The median OS of all LM patients was 9.8 months. The median OS of LM after BM, concurrent BMLM, and LM only was 8 months (95% CI= 2.58–13.56), 41.5 months (95% CI= 0.00–94.36), and 18.5 months (95% CI=3.68–33.32), respectively. Multivariate Cox regression analysis showed only IT MTX (p= 0.010, HR= 0.392, 95%CI= 0.19–0.80) was associated with survival.

**Conclusion:**

MDT in the TKI era has led to a dramatic improvement of OS in patients with LM (4–6 months vs. 9.8 months). NSCLC patients with LM only and concurrent BM LM has a better prognosis and longer survival, and thus are worth receiving intensive MDT care.

